# Inferring Diversity and Evolution in Fish by Means of Integrative Molecular Cytogenetics

**DOI:** 10.1155/2015/365787

**Published:** 2015-08-09

**Authors:** Roberto Ferreira Artoni, Jonathan Pena Castro, Uedson Pereira Jacobina, Paulo Augusto Lima-Filho, Gideão Wagner Werneck Félix da Costa, Wagner Franco Molina

**Affiliations:** ^1^Departamento de Biologia Estrutural, Molecular e Genética, Universidade Estadual de Ponta Grossa, 84030-900 Ponta Grossa, PR, Brazil; ^2^Departamento de Zoologia, Universidade Federal de Pernambuco, Cidade Universitária, 50670-420 Recife, PE, Brazil; ^3^Instituto Federal de Educação, Ciência e Tecnologia do Rio Grande do Norte, 59500-000 Macau, RN, Brazil; ^4^Departamento de Biologia Celular e Genética, Centro de Biociências, Universidade Federal do Rio Grande do Norte, 59078-970 Natal, RN, Brazil

## Abstract

Fish constitute a paraphyletic and profusely diversified group that has historically puzzled ichthyologists. Hard efforts are necessary to better understand this group, due to its extensive diversity. New species are often identified and it leads to questions about their phylogenetic aspects. Cytogenetics is becoming an important biodiversity-detection tool also used to measure biodiversity evolutionary aspects. Molecular cytogenetics by fluorescence *in situ* hybridization (FISH) allowed integrating quantitative and qualitative data from DNA sequences and their physical location in chromosomes and genomes. Although there is no intention on presenting a broader review, the current study presents some evidences on the need of integrating molecular cytogenetic data to other evolutionary biology tools to more precisely infer cryptic species detection, population structuring in marine environments, intra- and interspecific karyoevolutionary aspects of freshwater groups, evolutionary dynamics of marine fish chromosomes, and the origin and differentiation of sexual and B chromosomes. The new cytogenetic field, called cytogenomics, is spreading due to its capacity to give resolute answers to countless questions that cannot be answered by traditional methodologies. Indeed, the association between chromosomal markers and DNA sequencing as well as between biological diversity analysis methodologies and phylogenetics triggers the will to search for answers about fish evolutionary, taxonomic, and structural features.

## 1. Introduction

Investigating 50% of the total biodiversity is a hard and exciting task for most ichthyologists. Fish are represented by 32,900 species; more than 20,000 are marine and 8,000 are living in Neotropical continental waters [1, 2].

Cytogenetics, which is the study of chromosomes, is becoming an important biodiversity-detection tool also used to measure biodiversity evolutionary aspects [3, 4]. Cytogenetics also enables the development of evolutionary, taxonomic, and phylogenetic inferences resulting from the support provided by the conservation of Neotropical fish species [5]. Firstly, this scientific field took steps towards describing and defining the chromosomal morphology of the species according to usual cytogenetic analysis methods such as the conventional staining. The first marks in the chromosome of fish were identified by classic chromosome techniques, C-banding, and the detection of nucleolar organizing regions (Ag-NORs); for review see [6]. The identification marks revealed much information about the evolutionary processes within this group such as chromosome rearrangements, structural and/or numeric polymorphisms, and sexual chromosome systems and variations associated with the geographic distribution of some species and/or populations [4, 7]. Although these techniques have enabled good insights into the understanding of chromosome diversity in fish, the access to the genome was limited, mainly in many families that have quite stable conservative karyotypes and banding patterns, a fact that has hindered the detection of their most diverse genetic divergence levels [8].

The methodological advances on chromosome analyses drastically grew in the last decades; they showed refinement and more accurate resolution power, due to molecular cytogenetics by fluorescence* in situ* hybridization (FISH) [9]. Such technique strongly improved the transition from classic to molecular cytogenetics. It allowed integrating the quantitative and qualitative data from DNA sequences and their physical location in chromosomes and genomes [10]. The FISH technique enables identifying the DNA sequences in the studied cytological material (target DNA), no matter if they are chromosomes or interphase nuclei fixed on the surface of the slide.

Many changes in this technique have been adapted and improved in cytogenetics laboratories, due to advances in microscopy and bioinformatics. However, FISH principles and procedure steps (marking, hybridizing, and detecting) have remained the same. Its greater resolution power plays an important role in precisely featuring the chromosome structures of many fish species [11]. Different technique types, based on the herein referred methodology, such as genome* in situ* hybridization (GISH),* in situ* simultaneous location of different chromosome regions by Multicolor FISH (m-FISH), and identification of specific regions by chromatin fiber extended (Fiber-FISH) or spectral karyotype (SKY), are known and applied to distinct means.

The mapping of repeating DNA has been the main use of the FISH applied to fish. Among these sequences, it is possible to highlight the chromosome locations of multigene families such as 18S and 5S ribosomal DNA, histones, telomeric and centromeric sequences, and transposable elements [11]. The isolation and application of specific satellite DNA probes have also been the routine method in intraspecific characterization as well as in the understanding of evolution sequences among related species [12]. These probes help detecting chromosomal homeologies by identifying syntenic groups kept or rearranged during karyotype divergence among analyzed species [13]. These studies aim to understand the structural nature and the likely origin of B or supernumerary chromosomes [14] and to draw the origin and evolution of sexual chromosomes [15] and their behavior in the meiotic cells [16].

FISH potentialities are highly broad. The technique enables the better grounding of hypotheses heading towards structural, taxonomic, population, and/or phylogenetic aspects. It happens along with the easiness and availability of prospecting probes through different strategies [17]. These data are crucial for understanding the chromosomal dynamics and evolution and how it may be linked to speciation processes and macroevolutionary events. Throughout the data presentation, the current study aims to assess the contributions provided by the herein described technique and to integrate fish genome.

## 2. Cryptic Species Detection and Population Structuring in Marine Environments

Marine biogeography studies the history of marine taxa geographic distribution and it aims to set endemism areas as well as broad global distribution patterns [18]. The combined action of certain parameters such as the pelagic larval period duration, the individual's power to disperse in adulthood, and the actions resulting from ocean currents have shaped the chromosomal diversity of species, although it is difficult to indicate one single determining factor of cladogenetic events.

There is an exuberant diversity of shapes and wide body size variety in the Perciformes order, which holds more than 10,000 species [19]. This morphological diversity contrasts with the high karyotype stability [20] found in most representatives of this order. They are acrocentric diploid and have fundamental number 2*n* = 48 chromosomes, simple ribosomal sites, and little heterochromatin mostly concentrated in the pericentromeric regions [21]. However, the stronger chromosomal dynamism is shaped by pericentric inversions and, in smaller proportion, by centric fusions, just as it happens in some families, mainly in those that live in coral reefs [22]. These evidences enable discriminating populations in the Brazilian coast by classic banding [23], although such procedure is not always resolute.

Studies focused on population cytogenetics are incremented by the use of* in situ* hybridization. FISH was used to discriminate so far nondiagnosed cryptic karyotypes by conventional techniques. Indeed, its use detected different fishery stocks of the circumtropical species* Caranx lugubris* in São Pedro and in São Paulo Archipelagos (1,100 Km away from the Brazilian coast). Although they apparently present homogeneous karyotypes regarding chromosomal morphology, C-banding, and Ag-NORs, these morphotypes diverged on the frequency of 5S ribosomal gene sites, thus corroborating the distinct morphological patterns. These data suggest that the possible contact among stocks from other oceanic regions may take place in these oceanic islands [24]. This same analytical approach also shows population fragmentation in* Bathygobius soporator* within the Northeast region of Brazil as well as revealing remarkable chromosomal differentiations among coastal populations and populations in Rocas Atoll, regions approximately 267 Km geographically distant from each other, a fact that points towards new species in the area [25].

**Figure 1:**
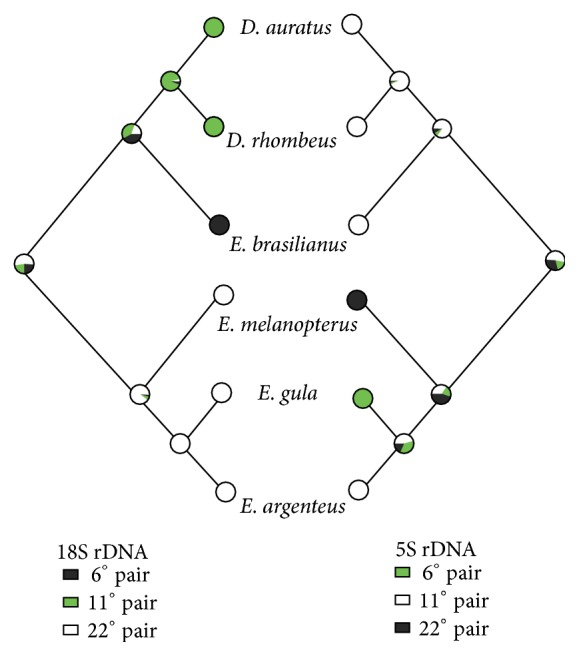
Reconstruction of ancestral characters of 18S and 5S rDNA in six species from the Gerreidae family (*Eucinostomus gula, Eucinostomus melanopterus, Eucinostomus argenteus, Diapterus auratus, Diapterus rhombeus*, and* Eugerres brasilianus*), which was gotten by means of the Mesquite software using the MK-1 model. Phylogenetic hypothesis estimated by likelihood analysis based on mtDNA COI sequences.

The use of available molecular phylogenies enables associating chromosomal patterns and preestablished topologies. It allows follow-ups in the history of these characters. The interaction among DNA sequences and chromosomes enables polarizing the chromosomal changes, identifying the consistency of phylogenetic signs or the possible homoplasies and evolutionary trends, and diversifying mechanisms in the group [26]. Drawing the history of these characters seems to be a big challenge in Perciformes, since the established chromosomal conservatism strongly features many families [21]. Actually, this trend is found in the Gerreidaefamily, which shows most of its representatives with 2*n* = 48 acrocentric chromosomes [27, 28]. It is possible to identify the independent evolution of these genes in both genera,* Diapterus* and* Eucinostomus *(Figure 1), by crossing the history of the characters and the information from the 18S and 5S ribosomal genes. Whereas the 18S rDNA sites present higher location variability in* Diapterus* [28], this same gene, in* Eucinostomus*, appeared to be quite conservative and located in the same pair of different species of this genus [27]. On the other hand, inverse pattern is found in 5S rDNA sites, in which the loci are located in different pairs in representatives of the genus* Eucinostomus*, whereas, in* Diapterus*, this same genus appeared to be conservative in the analyzed species [27, 28].

## 3. Intra- and Interspecific Karyoevolutionary Aspects of Freshwater Groups

Freshwater fish offer extremely informative models to investigate geologic background and connectivity among basins during the identification of biogeographic processes [29]. The Neotropical region offers excellent opportunities to the study on diversification mechanisms among freshwater fish. The region holds the biggest biodiversity in the world due to complex ecological and historical processes that deal with isolation and specialization [30]. More than 1,000, out of the 8,000 estimated species, already have information about their karyotype and demonstrate great diversity in diploid values. It covers from 2*n* = 20 chromosomes in* Pterolebias longipinnis* (Rivulidae) up to 2*n* = 134 in catfish* Corydoras aeneus* [31].

Continental systems physical subdivisions are often effective in blocking gene flow. Such condition leads to species endemism in some regions [32], whereas, in others, despite demanding broad contribution, they are morphologically categorized as a single taxon. They show remarkable differences in the chromosomal number as well as showing morphology that highlights the occurrence of complexes of species [4, 33].

*Hoplias malabaricus* consists of seven karyomorphs. It provides an exceptional way to understand historical relations in different draining within the Neotropical region [4, 34]. Close relations are found among populations from adjacent basins that share the same karyomorph and they indicate the phylogenetic ancestry among these hydrographic basins. However, differences in the number of 18S ribosomal sites point towards historical gene flow restrictions among these populations [35]. The mapping analysis of 5S*Hind*III satellite DNA is effective in characterizing allopatric populations of such species in DNA sequence mapping when there is no divergence between pairs carrying the 18S rDNA. It shows clear interpopulation divergences that result from different evolutionary histories triggered by this basin's geological isolation [13].

The taxonomic relations in the Parodontidae family, which presents stable karyotypes, are the target of controversies [36]. Although the location of 18S ribosomal genes appears to be discriminating between the genera* Parodon* and* Apareiodon*, the first genus possesses more conservative conditions. The first ones show more conservative conditions located in the terminal region of the long arm of one subtelocentric chromosome pair in all species studied thus far and in* Parodon* it reveals higher dynamism among the species [37]. The 5S rDNA sites appear to be more conservative in Parodontidae, found in pericentromeric position in a submetacentric pair similar in Anostomidae, sister family [38].

The mapping of 5S rDNA enables discriminating sister-species such as* Oligosarcus solitarius* and* O. argenteus* (Characiformes). The complementary localization data, in 18S rDNA sites, allow interpopulation differentiations in* O. solitarius* [39].

Briefly, for instance, the countless FISH applications through 18S and 5S ribosomal genes appear to be resolute in establishing phylogenetic relations in species discrimination as well as in understanding population historical relations in either freshwater or marine environments.

## 4. Evolutionary Dynamics of Marine Fish Chromosomes

Some fish groups, mostly in the Perciformes order, show a well-known low karyotypes dynamics [8]. Overall, such condition is due largely to biological and environmental characteristics of the marine biome. Species with high dispersive potential [23] and high population contingents [40] seem to be particularly refractory to chromosomal change fixations in their karyotypes. These conditions are particularly pronounced in marine species. The absence of geographic barriers and the attention given to the other two conditions are more easily found.

**Figure 2:**
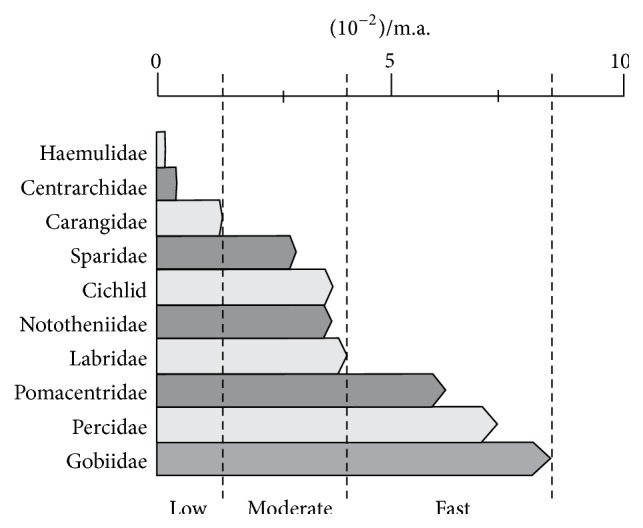
Karyotype diversification taxa in some Perciformes families (modified by Molina et al., 2014). Groups presenting low, moderate, and fast chromosomal divergence rates allow differentiated uses of FISH mapping in the analyses of their evolutionary dynamics.

Karyotype evolution rates in Perciformes can be classified in three diversification levels, that is, low, moderate, and high. Some families present bradytelic evolution, which is once more exemplified by taxa as low as 0.094 × 10^−2^/m.a. in Haemulidae. There is a second group, which holds families that present moderate karyotype evolution, horotelic, and in which Sparidae and Labridae show rates that, respectively, vary from 2.933 × 10^−2^/m.a. to 4.031 × 10^2^/m.a. There is one last group composed of families such as Pomacentridae and Gobiidae that present remarkable tachytelic evolution with rates that vary from 6.124 × 10^−2^/m.a. to 8.943 × 10^−2^/m.a. The karyotype evolution rate varies in the range of one hundred times (Figure 2). Perciformes constitute an exceptional evolutionary model for chromosomal studies [41].

It is possible to find some families of marine Perciformes such as Sciaenidae, Chaetodontidae, Gerreidae, and Lutjanidae [23, 27, 41, 42] among groups that show slow karyotype evolution. These groups present karyotype composed of 2*n* = 48 acrocentric chromosomes and it is a plesiomorphic condition shared by Percomorpha [43]; thus it possibly reaches Acanthopterygii [41].

Entire families can share the same karyotype, but it is practically unchangeable under the sieve of classic cytogenetic analyses. As for Lutjanidae, the so far analyzed species often present common karyotype (2*n* = 48 acrocentric chromosomes). This is the same condition found, for instance, in Sciaenidae, Chaetodontidae, Haemulidae, and Gerreidae [44]. Such karyotypes share features such as symmetric chromosomes (small size difference among bigger and smaller karyotype elements), reduced heterochromatic regions, homogeneous heterochromatin [40], and single ribosomal sites (Ag-NORs) [21]. According to an evolutionary perspective, these chromosomal characters significantly hinder the establishment of intraspecific diversity indicators as well as of phylogenetic inferences among the species.

The Ag-NORs sites are effective cytotaxonomic loci in conservative karyotypes. Approximately 330 teleost species from 77 families distributed into 22 orders were already analyzed. NORs present single sites in 72% of the species; besides, they constitute notably diverse regions in comparison to other genes [45]. Thus, in some cases, rDNA may appear limited to identify exclusive differentiations in its positioning and frequency, mostly among species from families that present notable chromosomal conservatism and, therefore, low evolutionary dynamics [40, 41].

Despite the fact that cytogenetic analyses provide more robust data, their exclusive use is quite limited and it just covers a short percentage (1.3%) of teleost species [45]. The* in situ* DNA sequence mapping enables identifying high syntenic chromosomal conservatism among species from marine fish families [40]. It shows that it can be an extensive condition to have a broad spectrum in one single clade. On the other hand, in some cases, it also enables evidencing the occurrence of extensive evolutionary changes with regard to the dispersion of some repeating sequences, both coding or noncoding.

Multigene families are repeating sequences of coding DNA that belong to a family of related proteins coded by a set of similar genes. These families are formed by duplication events during evolution. The observed differences reflect the mutations that took place throughout time. Histone and ribosomal genes are found among these differences. Chromosomal rearrangements disperse the multigenic families through the genome, which may be followed by the physical mapping of its sequences. Generally, the multigene families present a considerable number of pseudogenes; thus they show similarities with functional genes in the same family, although they enable functionality due to the acquired mutations [46].

rRNA genes are among the better known multigene families in fish. In fact, the 18S and 5S ribosomal genes are the highlighted repeating sequences mostly found in fish chromosomal evolutionary studies [45].

**Figure 3:**
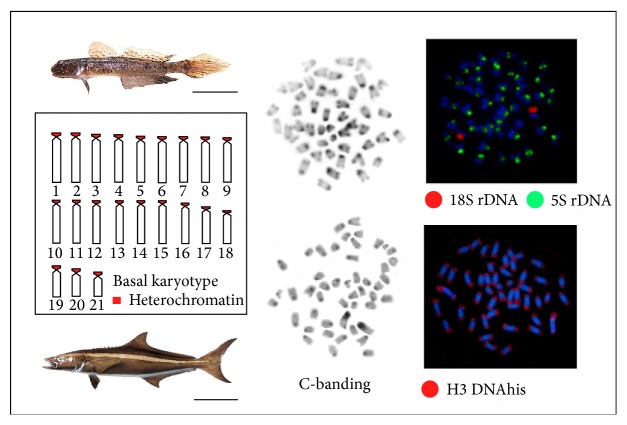
Basal karyotype pattern in the Perciformes and extensive dispersion events of sequences of the 5S DNAr multigenic families in* Ctenogobius smaragdus* (above) and of DNAhis H3 in* Rachycentron canadum* (below). Bar = 1 and 10 cm, respectively.

The evolution of 5S rDNA genes is progressively better understood [47]. The molecular variability observed for 5S rDNA gene is due mainly to NTS regions (nontranscribe spacer regions). Retrotransposons and microsatellites also seem to be involved with the high dynamism of their sequences [48]. The 5S rDNA is distributed into one or a few sites [46], among various fish groups. However, the physical mapping of these sequences by FISH in rare situations has revealed a massive dispersion in most of the chromosomes of some species. Such situations are observed in families with conservative evolutionary patterns such as Pomacanthidae [49] as well as in those with more dynamic patterns such as Gobiidae [25]. In this last family, the species* Ctenogobius smaragdus*, with 2*n* = 48 chromosomes, exhibits 5S hybridization marks in 42 chromosomes of the karyotype (Figure 3), and it suggests that not all these regions are active [50].

The complementary mapping of the 18S and 5S ribosomes enables the better and more efficient option of specific cytotaxonomic loci in some groups of species with high karyotype conservatism. As for Haemulidae, which is a group with genera that show low evolutionary dynamics (e.g.,* Haemulon*,* Pomadasys*, and* Conodon*), the species share a uniform karyotype [40, 51]. Species carrying symmetric karyotypes formed by acrocentric chromosomes with reduced heterochromatic content and, in general, with single ribosomal sites present limitations to identify interspecific diversification patterns by means of classic cytogenetic methods. Indeed, as for these cases, the combined use of mapping by applying the double FISH to 18S and 5S genes is particularly indicated. It enables analyzing these genes' genomic dynamics as well as identifying the interspecific diversity [27, 28].

With regard to other marine fish groups, the physical map of H1, H2B, H2A, and H3 histone genes shows interesting information about their evolution and it leads to insights into their dynamics in the species' karyotype [25, 50]. These genes play a key role regarding changes in the chromatin structure, in cellular cycle progression, and in gene activity repression [52, 53]. Besides, little is known about their physical positioning in the chromosome of fish. The H1 multigenic family constitutes the histone class with the faster evolutionary rate, whose diversification, mainly by evolution in concert [54], has been questioned in favor of birth-and-death evolution [55].

Cytogenetic analyses among populations and* Bathygobius* (Gobiidae) species, which is a group with high evolutionary dynamism, showed that H1 histone genes positioning and frequency are conserved with two sites kept in homologs chromosomes [25]. On the other hand, the H2B-H2A DNAhis and H3 DNAhis genes in* Rachycentron canadum* (Rachycentridae) presented the more diversified condition. Actually, this species of H2B-H2A genes presented multiple sites distributed in up to 6 chromosome pairs in the karyotype, whereas H3 sequences (Figure 3) are surprisingly distributed throughout all the chromosome pairs [50]. These data suggest a diversification pattern that matches the birth-and-death evolutionary model. It is followed by the purifying selection found in H2B-H2A histone genes as well as by the variation resulting from the evolution in concert, which involves H3 histone genes. These data reinforce the independent evolution of histone genes in this species. These genes appear to be colocated with many other repetitive DNA, microsatellites, 18S and 5S rDNA, transposons, and retrotransposons [42]. Distribution analysis of H3 DNAhis site, in five Lutjanidae species, showed that these genes are found in a single site in most of species (*Lutjanus analis, L. synagris,* and* L. alexandrei*). However, they can be found in two loci (*L. jocu*) or extensively dispersed in 44 out of the 48 chromosomes in* Ocyurus chrysurus* [42]. This low diversification pattern, which is highlighted by significant changes in the dispersion of sequences, is associated with the participation of transposable elements in the species' karyotype [50].

Repetitive DNA represent approximately 50% of the eukaryote genomes. The accumulation of these repetitive sequences is responsible for the variation in the genome size in the eukaryotes. It points out their influence on DNA replication, recombination, and gene expression band on the differentiation of sexual and B chromosomes [56]. Besides, the repetitive DNA are also involved with chromosomal rearrangements such as deletions, duplications, inversions, and reciprocal translocations, thus providing karyotype diversification in many groups [57]. The physical mapping of these sequences in the chromosomes enables accessing countless aspects related to the origin and evolution of sexual and B chromosomes [58, 59].

## 5. The Origin and Differentiation of Sexual and B Chromosomes

Another main element in the karyotype structure of fish regards the presence of sexual chromosomes. Fish, differently from birds and mammals, do not present sexual chromosomes on the basis of their phylogeny. Thus, this character independently and repeatedly emerged in the evolutionary history of this group. Some species present quite differentiated sexual chromosomes, although such occurrence is not frequent.

Sex chromosomes systems with female heterogametic (ZZ/ZW) are found in some species in the genera* Leporinus* [60],* Parodon* [61], and* Triportheus* [62]. It is evident, in most of these cases, that sex chromosome differentiation was followed by the heterochromatinization process and by changes in the size of W chromosome. XX/XY systems were described in* Pseudotocinclus tietensis* (Loricariidae) and also in the genus* Hoplias* [63], respectively. In addition, simple systems (XY and ZW) may undergo rearrangements and originate multiple sexual systems. There is the case of multiple sex chromosomes with female heterogamety (ZZ/ZW_1_W_2_) reported in* Apareiodon affinis* [64].

The systems with male heterogamety have the first records in Neotropical fish in the genera* Hoplias,* with multiple sex chromosomes such as X_1_X_1_X_2_X_2_/X_1_X_2_Y and XX/XY_1_Y_2_ [65], and* Eigenmannia,* with a X_1_X_1_X_2_X_2_/X_1_X_2_Y system [66].

So far, the chromosomal location of these genes in most fish species is still an open issue, although sex-determining genes may occur even without the presence of morphologically differentiated sex chromosomes. On the other hand, consistent advances have been achieved in the detection of these sexual chromosomes as well as in the origin and differentiation processes, by applying the whole chromosome painting (WCP). Some examples well depict this situation either in simple or multiple systems. Within simple systems, it is possible to identify the genes differentiation discerning homologous segments among the allosomes, as it can be seen in species in the genus* Triportheus* [67]. In multiple systems, the origin of sex neochromosomes is evidenced by Robertsonian fusions, as it is seen in species in the genus* Harttia *[68]. Furthermore, the use of chromosome painting also helps solving issues of common and/or independent origin such as the case linked to the Erythrinidae family in which the multiple system X_1_X_1_X_2_X_2_/X_1_X_2_Y found in* Hoplias malabaricus* and* Erythrinus erythrinus* presented independent origins [59].

Using FISH to investigate sex chromosomes is also a way to determine the location of ribosomal genes related to heterogametic sex chromosomes differentiation. There is an example of it in the W chromosome from the ZW system in genus* Triportheus* [62] and from the XX/XY system in* Hoplias* [35]. Besides, it has been possible to detect the residual interstitial telomeric sequences (ITS) from the centric fusion process in the formation of the XX/XY_1_Y_2_ multiple sexual chromosome system in* Harttia carvalhoi* [68]. Heterochromatization processes played an active role in the process of differentiating diverse sexual chromosome systems. The location of repetitive sequences over the sexual chromosomes helps elucidating differentiation processes due to the presence of* Rex* elements spread over the W chromosome in* Semaprochilodus taeniurus* [69] and the presence of microsatellites and different* Rex* in the Z and W chromosomes in* Triportheus trifurcatus* [15].

**Figure 4:**
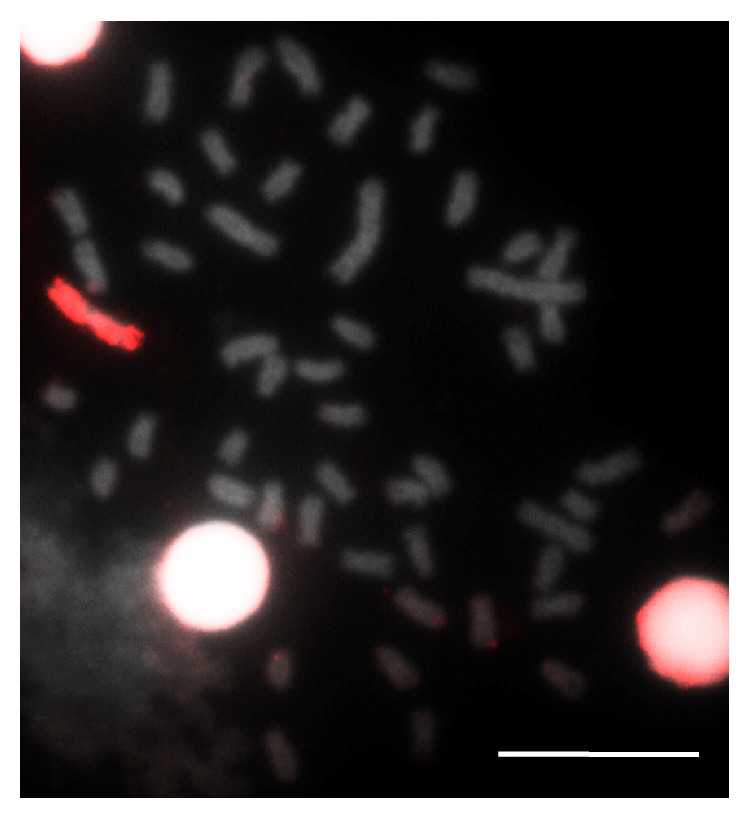
*Astyanax scabripinnis* metaphase chromosomes subjected to WCP with B chromosome probe amplified by DOP/PCR and marked by nick translation with streptavidin (red). Bar = 10 *μ*m.

Another emblematic matter concerning the karyotype of fish regards the occurrence of B or supernumerary chromosomes, as it is observed in* Apareiodon piracicabae* and* Paraligosarcus pintoi* [70],* Prochilodus lineatus* and* P. cearensis* [71],* Curimata modesta* [72],* Steindachnerina insculpta *[73],* Schizodon* [74], among others. Those are additional chromosomes that do not recombine with those from the standard karyotype complement and follow their own evolutionary path [75]. Among fish, it is possible to highlight the complex of* Astyanax scabripinnis* species as the currently more studied model concerning supernumerary chromosomes (Figure 4) distribution, behavior, and origin (for review check [76]).

**Figure 5:**
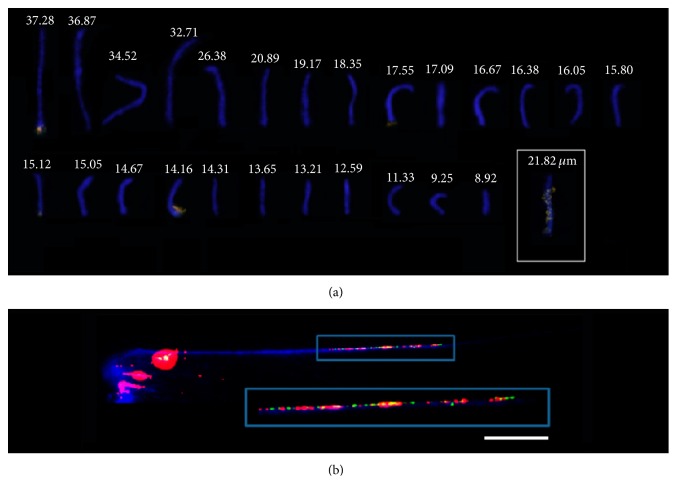
(a)* Astyanax scabripinnis* pachytene elements subjected to double FISH with 18S ribosomal DNA probe (green) and those of the* As*51 repetitive sequence (red). These sequences are interspaced, showing syntenic and syntopic location, and present highlighted broad B chromosome distribution. The total length of each element is expressed in *μ*m. (b) Detail of the extended chromatin fiber subjected to double FISH showing the syntenic and syntopic colocation of 18S and* As*51 sequences. Bar = 10 *μ*m.

The use of probe* As*51 from repetitive DNA over the chromosomes, gotten by means of total DNA cut with the* Kpn*I restriction enzyme, within species from the complex* A. scabripinnis, *identified its association with the 18S ribosomal DNA and the heterochromatic regions located in different autosomes and in the B chromosome [77]. These data reinforce previous hypotheses [78] about the possible intragenic origin of B chromosome in* A. scabripinnis* due to the formation of isochromosome in the standard complement. Actually, recent evidences corroborate B chromosome autopairing in the pachytene of meiotic cells in* A. scabripinnis* by applying FISH with* As*51 and WCP with B chromosome probe [14], gotten by microdissection and amplification by degenerate nucleotide primed and polymerase chain reaction (DOP-PCR) (Figure 5).

## 6. Conclusions and Further Perspectives

Fish might compose the biggest and most exciting challenge in the karyoevolutionary studies among vertebrates. No doubt that any other group rivals fish's huge biological diversity, which results from historically differentiated evolutionary processes that lead to their fascinating karyotype diversity. The diffusion of molecular biology tools provided great advance to the chromosomal study in fish, in addition to the classic staining methodology and chromosomal banding. Thus, the fluorescent* in situ* hybridization (FISH) enables evolutionary analyses resulting from the location of moderately (e.g., 18S rDNA, 5S rDNA, and histones) and highly repetitive DNA sequences (satellite DNA, transposable elements, microsatellites, and heterochromatin) over the chromosomes.

Variations in the FISH procedure are rarely applied to fish. Such technical bottleneck affects the understanding of fish chromosome structure by restricting evolutionary inference possibilities since most of the species do not present clearly defined structural longitudinal bands (G-band and R-band).

This new cytogenetic field, called cytogenomics, is spreading due to its capacity to give resolute answers to countless questions nonaccessible by traditional methodologies. Indeed, the association of chromosomal loci with DNA sequencing and of biological diversity analysis methodologies with phylogenetics triggers the will to search for answers regarding evolutionary, taxonomic, and structural matters in fish. The most recent expectation lies in overcoming technical impairments regarding the use of FISH's varying techniques (WCP of euchromatic regions and mainly micro-FISH) as well as in the new integration with the up-to-date next-generation sequencing (NGS) techniques to be applied to this fantastic group of animals.
